# Insecticide-Degrading *Burkholderia* Symbionts of the Stinkbug Naturally Occupy Various Environments of Sugarcane Fields in a Southeast Island of Japan

**DOI:** 10.1264/jsme2.ME14124

**Published:** 2014-12-27

**Authors:** Kanako Tago, Takashi Okubo, Hideomi Itoh, Yoshitomo Kikuchi, Tomoyuki Hori, Yuya Sato, Atsushi Nagayama, Kentaro Hayashi, Seishi Ikeda, Masahito Hayatsu

**Affiliations:** 1 Environmental Biofunction Division, National Institute for Agro-Environmental Sciences3–1–3 Kannondai, Tsukuba, Ibaraki 305–8604Japan; 2 Bioproduction Research Institute, National Institute of Advanced Industrial Science and Technology (AIST) Hokkaido2–17–2–1 Tsukisamu-higashi, Toyohira-ku, Sapporo, Hokkaido 062–8517Japan; 3 Research Institute for Environmental Management Technology, AISTTsukuba, Ibaraki 305–8569Japan; 4 Okinawa Prefectural Agricultural Research CenterItoman, Okinawa 901–0336Japan; 5 Memuro Research Station, Hokkaido Agricultural Research Center, National Agriculture and Food Research Organization9–4 Shinsei Minami, Memuro-cho, Kasai-gun, Hokkaido 082–0081Japan

**Keywords:** *Burkholderia*, organophosphorus insecticide degradation, symbiotic gut bacteria, *Cavelerius saccharivorus*, sugarcane

## Abstract

The stinkbug *Cavelerius saccharivorus*, which harbors *Burkholderia* species capable of degrading the organophosphorus insecticide, fenitrothion, has been identified on a Japanese island in farmers’ sugarcane fields that have been exposed to fenitrothion. A clearer understanding of the ecology of the symbiotic fenitrothion degraders of *Burkholderia* species in a free-living environment is vital for advancing our knowledge on the establishment of degrader-stinkbug symbiosis. In the present study, we analyzed the composition and abundance of degraders in sugarcane fields on the island. Degraders were recovered from field samples without an enrichment culture procedure. Degrader densities in the furrow soil in fields varied due to differences in insecticide treatment histories. Over 99% of the 659 isolated degraders belonged to the genus *Burkholderia*. The strains related to the stinkbug symbiotic group predominated among the degraders, indicating a selection for this group in response to fenitrothion. Degraders were also isolated from sugarcane stems, leaves, and rhizosphere in fields that were continuously exposed to fenitrothion. Their density was lower in the plant sections than in the rhizosphere. A phylogenetic analysis of 16S rRNA gene sequences demonstrated that most of the degraders from the plants and rhizosphere clustered with the stinkbug symbiotic group, and some were identical to the midgut symbionts of *C. saccharivorus* collected from the same field. Our results confirmed that plants and the rhizosphere constituted environmental reservoirs for stinkbug symbiotic degraders. To the best of our knowledge, this is the first study to investigate the composition and abundance of the symbiotic fenitrothion degraders of *Burkholderia* species in farmers’ fields.

Bacteria are known for their ability to occupy remarkably diverse ecological niches and frequently associate with plants and animals. Some environmental bacterial species can transmit into plant and animal tissues, forming a mutualistic symbiosis with their host. Bacterial symbionts are considered to promote host fitness, thereby supporting their own spread. The mechanisms underlying the environmental transmission of symbionts have been studied in some representative species (5, 12, 34). However, the ecological properties of symbiotic bacteria in free-living environments remain relatively uncharacterized.

The genus *Burkholderia* is widely distributed in terrestrial environments; members of this genus have been isolated from soil (32), the rhizosphere (8, 39), plants (3, 7), insects (4), fungi (35), and infected humans (see reviews in [9, 11, 40]). Some species of *Burkholderia* are symbionts of pest stinkbugs such as *Riptortus pedestris*, a notorious pest of leguminous crops in Eastern Asia, and its relative *Cavelerius saccharivorus*, a serious sugarcane pest in the subtropical region stretching from Taiwan to the Southwestern islands of Japan (20, 21, 23). Free-living symbiotic *Burkholderia* are transmitted from the environment to stinkbugs and colonize their symbiotic midgut organs called crypts (20, 22). Symbiotic *Burkholderia* promote host growth and reproduction (22, 26), strongly suggesting a pivotal role of the symbiont in host metabolism. Phylogenetic analyses previously revealed that symbionts commonly isolated from these insects formed a cluster designated “the stinkbug-associated beneficial and environmental (SBE) group” within the genus (20, 23).

In agricultural land, fenitrothion (*O*,*O*-dimethyl *O*-(4-nitro-*m*-tolyl) phosphorothioate) is widely used to control pests including stinkbugs. Some strains of *Burkholderia* species capable of degrading fenitrothion have been isolated from soil and sludge exposed to fenitrothion in China (45), Japan (18, 41), and South Korea (27). Because several fenitrothion degraders are phylogenetically closely related to the SBE group, the possibility of these degraders establishing a symbiotic relationship with *R. pedestris* was previously investigated using microcosms (24). A symbiotic relationship was successfully established, providing the host, *R. pedestris*, with increased fenitrothion resistance and enabling its survival even after exposure to this insecticide. Furthermore, a recent field study demonstrated that approximately 8% of *C. saccharivorus* present in sugarcane fields exposed to fenitrothion harbored fenitrothion degraders, which were identified as *Burkholderia* species (24). These findings suggested that the fenitrothion degraders of *Burkholderia* species, including stinkbug symbionts, established their populations in sugarcane fields, and also that transmission to the host may occur. However, degraders have only been identified in soil and sludge exposed to fenitrothion under laboratory conditions. Furthermore, most stinkbugs feed on the upper parts of plants (1). Therefore, it currently remains unclear where and how symbiotic degraders encounter their hosts in the fields.

The diversity and distribution of symbiotic degraders in free-living environments can play a crucial role in the symbiotic relationship with stinkbugs. We previously demonstrated that some strains of *Burkholderia* quickly developed the ability to degrade fenitrothion after repeated exposure to this insecticide using soil microcosms (41). However, the actual ecological characteristics of the degraders including the symbionts in agricultural fields need to be evaluated at a local scale. Therefore, we herein examined the diversity and abundance of the fenitrothion degraders of *Burkholderia* species in soil and on sugarcane grown under agricultural field conditions.

## Materials and Methods

### Field sites

The field sites used in this study were located on a raised atoll, Minami-Daito Island, located in the Philippine Sea (25°50′N, 131°14′) 360 km east of Okinawa Island, Japan. Soils in Minami-Daito Island are classified as Lateritic Red soil and Lateritic Yellow soil (30). Agricultural land, mainly sugarcane fields, covers approximately 60% of the total area (30.57 km^2^) of the island. Most of the sugarcane fields had been regularly treated with fenitrothion for years prior to this study.

### Sampling and isolation of fenitrothion degraders

**Fig. 1:**
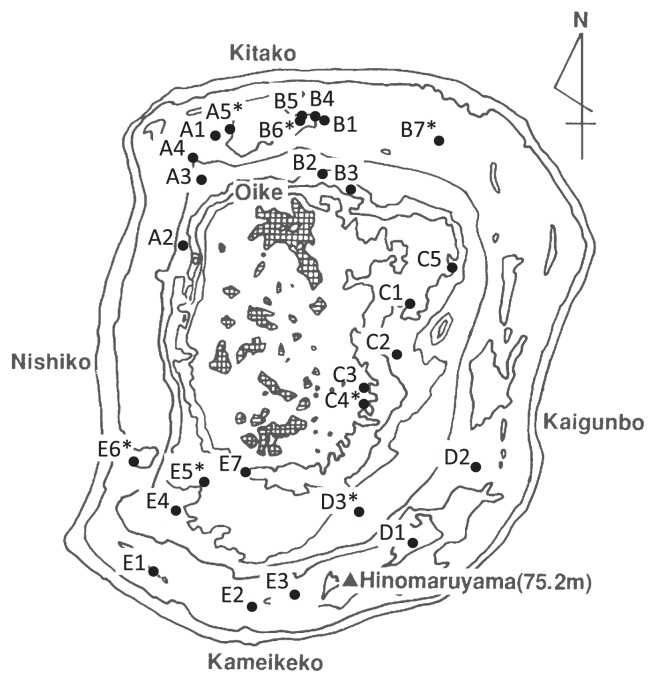
Map of sampling fields. Field numbers are indicated. The sampling fields in 2013 are represented by asterisks. A contour map is provided by the Geospatial Information Authority of Japan.

Soil samples were collected from 27 farmers’ sugarcane fields on the island in June 2010 (Fig. 1). Soil properties and insecticide treatment histories for the 2 years prior to sampling are listed in Table S1. Surface (0–1 cm) and subsurface (1–10 cm) soil samples were collected from furrows at three points in each field. The fenitrothion concentration appeared to be higher in surface soils than in subsurface soils owing to the adhesion of fenitrothion to the ground surface (42). The samples were sieved through a 2-mm pore size mesh and suspended in sterile water. To investigate the intrinsic community of degraders in these soil samples, the suspensions were serially diluted and directly plated onto a mineral salts agar medium containing 0.4 g L^−1^ yeast extract and 0.8 mL L^−1^ fenitrothion emulsion consisting of 50% fenitrothion (FNT medium) (18). Bacterial colonies visible on the FNT medium were counted after a 3-d incubation period at 30°C. Fenitrothion degraders were distinguishable based on the clear zones around the colonies owing to fenitrothion transformation or mineralization. The average numbers of CFU (colony forming units) g^−1^ dry soil of fenitrothion degraders were statistically analyzed by the Kruskal-Wallis test using SPSS Statistics Version 22 (IBM Japan, Tokyo, Japan) to evaluate the effects of insecticide treatments. Boxplots of the CFU data were produced using the boxplot function in R 3.1.0 (http://www.r-project.org) using the default parameters, except that outliers were not drawn. A *P*-value of less than 0.05 was considered significant.

Plant and rhizosphere samples were collected from seven sugarcane fields on the island (Fig. 1 indicated by asterisks) in May 2013. The fields were chosen based on the density of degraders, as evaluated in 2011 (mentioned below). The insecticide treatment history for a 2-year period prior to sampling is listed in Table S2. Samples of three sugarcane plants and their rhizospheres were collected from each field. The rhizosphere samples were sieved through a 2-mm pore size mesh and suspended in sterile water. The suspension was serially diluted and plated onto a *Burkholderia* semi-selective medium, *Pseudomonas cepacia* azelaic acid tryptamine medium (PCAT; 0.1 g L^−1^ MgSO_4_ , 2 g L^−1^ azelaic acid [sole carbon source], 0.2 g L^−1^ tryptamine, [main nitrogen source], 4 g L^−1^ K_2_ HPO_4_ , 4 g L^−1^ KH_2_ PO_4_ , 0.02 g L^−1^ yeast extract, 15 g L^−1^ agar [pH 5.7] (6)), containing 0.8 mL L^−1^ of 50% fenitrothion emulsion. Plant samples were washed with tap water to remove adherent soil particles and rinsed with sterilized water, and bacterial cells were isolated from both plant sections using a previously described method (19). Stem-associated bacterial cells were isolated from a stem section located 3–18 cm above ground level. Leaf-associated bacterial cells were isolated from a section containing the stem and leaf located 18–78 cm above ground level. The samples were homogenized in bacterial cell extraction buffer (50 mM Tris-HCl [pH 7.5], 1% Triton-X100, and 2 mM 2-mercaptoethanol) using a blender. The crude extract was filtered through a sterilized Miracloth (CalBiochem, La Jolla, CA, USA), and the filtrate was centrifuged at 500×*g* for 5 min at 10°C to remove plant residue. The supernatant was further centrifuged at 5,500×*g* for 20 min at 10°C, and then removed, and the pellet was resuspended in 50 mM Tris-HCl (pH 7.5). The cell suspension was serially diluted and plated onto PCAT medium. After a 3-d incubation period at 30°C, bacterial colonies with clear zones on PCAT medium were counted and purified.

### 16S rRNA gene sequencing analysis

Cell lysates of the purified fenitrothion degraders were prepared for PCR amplification of the 16S rRNA gene (41). PCR was conducted using the universal primers 27F and 1492R, and the PCR products were purified and directly sequenced using primer 519R as previously described (41). The 16S rRNA gene sequences were clustered into distinct types at 100% sequence identity. Nearly full-length 16S rRNA gene sequences (*ca.* 1400 bp) of the representative strains from each type were determined. Taxonomic assignment for the 16S rRNA gene sequences was conducted using BLASTN (2) against the DNA Data Bank of Japan (DDBJ) database. The types assigned as the genus *Burkholderia* were used for further analyses. The 16S rRNA gene sequences of the representative strains of the genus *Burkholderia* were subjected to a phylogenetic analysis with MEGA software version 6.06 (43). The sequences were aligned by the CLUSTAL W program (44) and the phylogenetic tree was constructed using the neighbor-joining method (37).

### Sampling and identification of stinkbugs

*C. saccharivorus* were collected from seven different sugarcane fields on Minami-Daito Island (Fig. 1, indicated by asterisks) in May 2013 and transported alive to the laboratory. After surface-sterilization with 70% ethanol, insects were dissected in a sterile PBS buffer to remove their symbiotic organ, the midgut fourth section, filled with their symbiotic *Burkholderia* (20). One half of the dissected organ was stored in 100 μL of fenitrothion buffer (0.2 mM fenitrothion, 50 mM Tris-HCl [pH 8.0], and 0.1% Triton X-100) and incubated at 27°C for 1 h without shaking (24). After the incubation, optical density at 405 nm derived from 3-methyl-4-nitrophenol, which is one of the hydrolytic products of fenitrothion (18), was measured using the ND-1000 spectrophotometer (NanoDrop Technologies, Wilmington, DE, USA). The remaining half of the dissected organ showing the hydrolytic activity of fenitrothion in the preceding assay was subjected to DNA extraction as described previously (23), and used as a template for the subsequent PCR amplification. A 1.5 kbp region of the bacterial 16S rRNA gene was amplified by PCR using AmpliTaq Gold polymerase (Applied Biosystems, Foster City, CA, USA) and a universal primer set for the bacterial 16S rRNA genes, 16SA1 and 16SB1 (16), under a reaction profile of 95°C for 9 min followed by 30 cycles of 95°C for 30 s, 55°C for 1 min, and 72°C for 1.5 min. Sequencing and the taxonomic assignment was conducted as described above.

### Degrader community analysis

The Yue and Clayton index measure of dissimilarity between the communities of fenitrothion degraders in the soil was estimated from the relative abundance of the types of degraders using Mothur version 1.33.3 (38). The resulting distance matrix was visualized by a principal coordinate analysis (PCoA). An analysis of molecular variance (AMOVA) was also conducted to evaluate the effects of insecticide treatments on the community patterns of fenitrothion degraders in soil. Differences among degrader communities in the sugarcane stem, leaf, and rhizosphere samples were also evaluated using the data from Fields A5, B7, and E6, because full datasets (including the stem, leaf, and rhizosphere) were available for these sampling sites.

### Accession numbers of nucleotide sequences

The 16S rRNA gene sequences of representative strains were deposited in the DDBJ databases under the accession numbers AB982930 through AB982967.

## Results

### Population density of fenitrothion degraders in sugarcane field soils

**Fig. 2:**
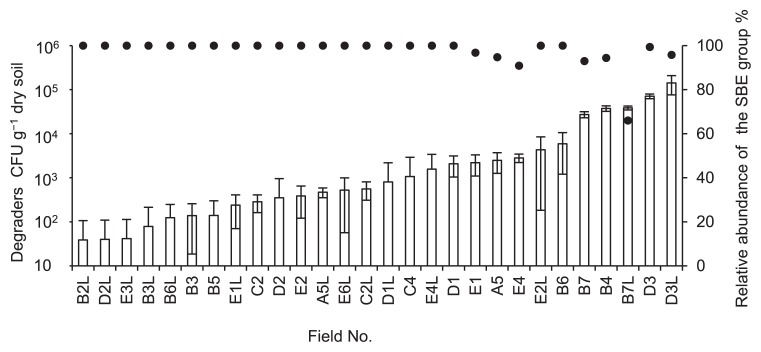
Degrader density (open bars) and the relative abundance of the SBE group (closed circles). If the sample was collected from subsurface soil, the subscript “L” is placed after the field number. Error bars indicate standard deviations (*n* = 3).

**Fig. 3:**
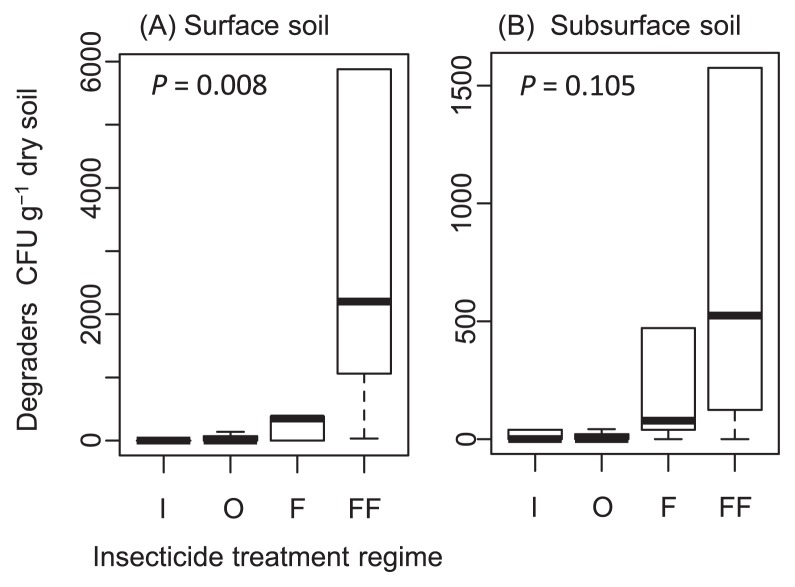
Box plot representing the CFU counts of fenitrothion degraders in surface (A) and subsurface (B) soil samples with different insecticide treatments. The insecticide treatment regimens are provided in Table S1. The boxes represent the range between the first and third quartiles (25th and 75th percentiles, respectively), and the inner line represents the median.

Fenitrothion degraders were recovered on FNT medium from either one or both layers (surface and subsurface) of soil in 17 out of 27 sugarcane fields. The density of the degraders varied among the soil samples up to 1.4×10^5^ CFU g^−1^ dry soil in Field D3 (Fig. 2, open bars). The effect of the insecticide treatment was statistically analyzed with respect to the colony count of the degraders (Fig. 3). The sugarcane fields had been subjected to different insecticide treatment regimens; the treatments selected for use in this study included a 2-year continuous treatment with fenitrothion in 2010 and 2011 (FF), a 1-year treatment with fenitrothion in 2010 or 2011 (F), a 1- or 2-year treatment with other organophosphorus insecticides (O), and both a 1- or 2-year treatment of non-organophosphorus insecticides and no treatment of insecticides (I) (Table S1, see “insecticide treatments” columns). The insecticide treatment significantly influenced the density of the degraders in surface soils (Kruskal-Wallis test, *P* = 0.008), but not in subsurface soils (*P* = 0.105) (Fig. 3). Among the surface soils, the density of fenitrothion degraders was significantly higher in FF soils than in O and I soils (*P* = 0.004 and *P* = 0.004, respectively), while no significant difference was noted between FF and F soils (*P* = 0.115). Degrader density was not significantly higher in F soils than in O or I soils (*P* = 0.355 and *P* = 0.243, respectively). These results indicated that degrader density in surface soils consistently correlated with the frequency of the fenitrothion treatment.

### Diversity of fenitrothion degraders in sugarcane field soils

**Fig. 4:**
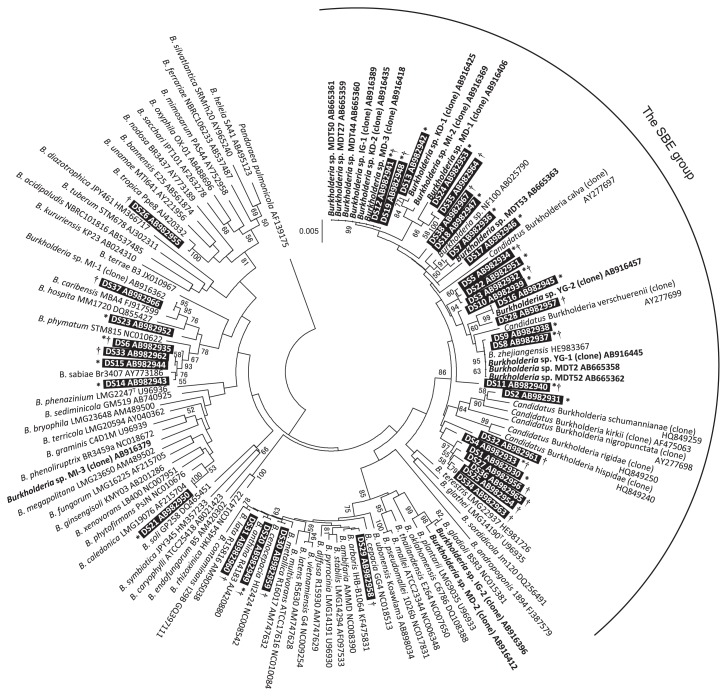
Phylogenetic tree of *Burkholderia* based on 16S rRNA gene sequences, showing the positions of reference strains and representative fenitrothion degraders from each of the 38 types. Representative degraders are designated ‘DS’ followed by the type number, and shaded black. Several known symbionts obtained from stinkbugs are represented in bold. The types isolated from soil samples collected in 2011 are indicated by asterisks. The types isolated from sugarcane and rhizosphere samples collected in 2013 are indicated by daggers. Accession numbers of reference sequences in the NCBI database are indicated. The 16S rRNA gene sequence of *Pandraea pulmonicola* AF139175 was used as an outgroup. Branch nodes with >50% supporting bootstrap values (1000 replicates) are shown.

**Fig. 5:**
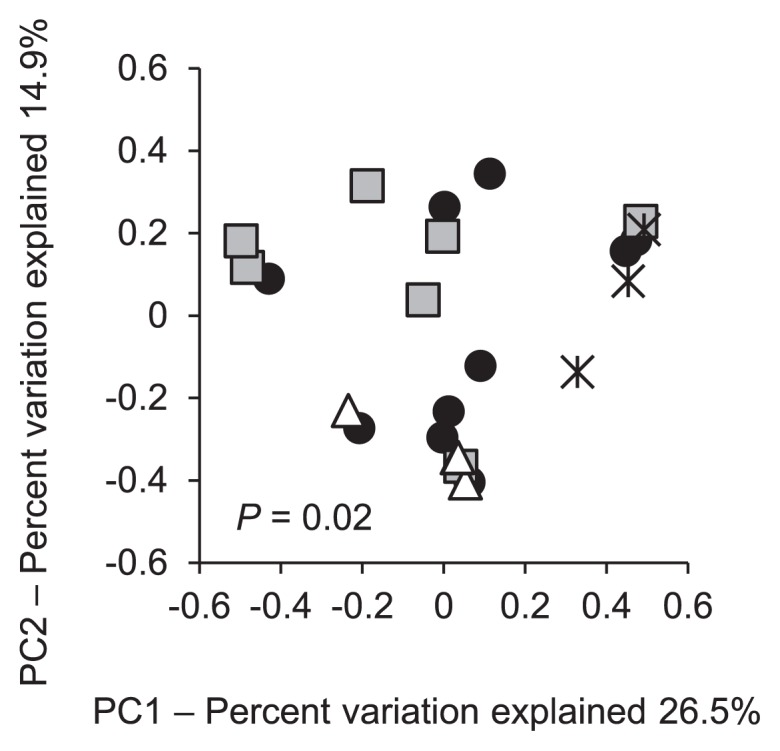
PCoA plots derived from pairwise distances between the communities of degraders in surface soils with different insecticide treatments. Symbols: closed circles, FF; grey squares, F; open triangles, O; asterisks, I. The insecticide treatment regimens are provided in Table S1.

Degraders in the soil samples were isolated and subjected to a 16S rRNA gene sequence analysis. In total, 657 out of 659 of the isolated degraders belonged to the genus *Burkholderia*; these were assigned to 24 distinct types (Type 1 through Type 24, Fig. 4 indicated by asterisks) based on 100% sequence similarity. The other two degraders belonged to the genera *Ochrobactrum* and *Pseudomonas*, respectively. Among the degraders of *Burkholderia* species, 18 types clustered with the SBE group (Fig. 4). Representative strains from each of these types were designated ‘DS’ followed by the type number in the phylogenetic tree (Fig. 4); for example, strain DS1 was the representative strain of Type 1. The degraders of the SBE group constituted the greatest proportion (66–100%) of the degraders in all soil samples (Fig. 2, closed circles); degraders outside the group were recovered at lower levels (up to 34%) from a subset of the soil samples, in which the degrader density was higher than 2.2×10^3^ copies g^−1^ dry soil (samples A5, B4, B7, B7L, D3L, E1, and E4 in Fig. 2). The PCoA analysis demonstrated distinctive trends in the community of the degraders in surface soils based on the insecticide treatment regime (*P* = 0.02) (Fig. 5).

The 16S rRNA sequencing analysis showed that representative strains of Types 8, 12, and 19 were 100% identical to the symbionts that had been found from *C. saccharivorus* living in sugarcane fields on the island (24). Type 8 was the most prevalent of the three, with a total of 107 isolates obtained from 13 fields (Table S3). Nineteen isolates of Type 12 and 34 isolates of Type 19 were obtained from 5 and 6 fields, respectively. This result strongly suggested that the soil-born fenitrothion degraders of *Burkholderia* species were transmitted to stinkbugs in agricultural fields.

### Degrader distribution in various niches

**Fig. 6:**
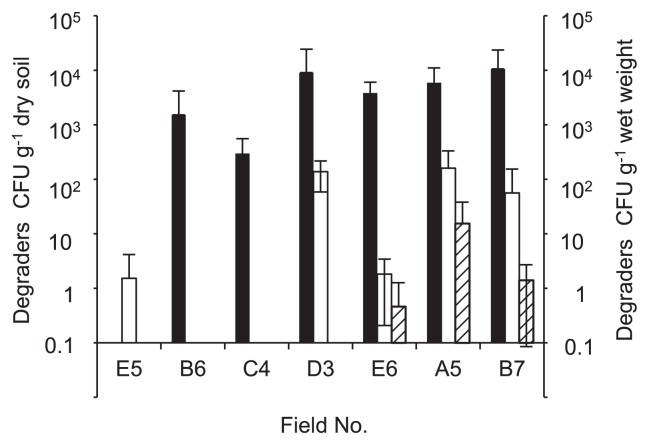
Degrader density in sugarcane stem (open bars), leaf (slash bars) and rhizosphere (black bars) samples. The detection limits of the rhizosphere and plant degraders were 100 and 0.1 CFU, respectively.

To investigate the diversity and distribution of the symbiotic fenitrothion degraders of *Burkholderia* species in fields at a local scale, seven fields (Fields A5, B6, B7, C4, D3, E5, and E6) were chosen based on the density of degraders evaluated in 2011 (Fig. 2). Sugarcane plant and rhizosphere samples were collected from the fields and used for analyses. Degraders were successfully recovered on PCAT medium from rhizosphere samples from the fields exposed to fenitrothion, except for Field E5, which had not been exposed to fenitrothion for two years prior to sampling (Fig. 6). The degrader density in the rhizosphere ranged from 3.0×10^2^ to 1.0×10^4^ CFU g^−1^ of dry soil. Degraders were recovered from sugarcane stem samples in the fields treated with fenitrothion at a high frequency (Table S2). Their densities ranged from 2 (Field E6) to 138 CFU g^−1^ wet weight (Field D3), which was less than 10% of that observed in rhizosphere samples (Fig. 6). Degraders were isolated at a low density from sugarcane leaf samples (1 to 15 CFU g^−1^ wet weight) (Fig. 6). To compare the communities of degraders present on the sugarcane plants (*i.e.* stem and leaf) and in the rhizosphere, degraders were isolated from samples from Fields A5, B7, and E6, in which degraders were detected in all three environments (stem, leaf, and rhizosphere), and were subjected to a phylogenetic analysis of 16S rRNA gene sequences. This analysis revealed that the isolates could be assigned to 25 distinct types belonging to the genus *Burkholderia*, with 361 out of 393 isolates clustering within the SBE group (Table 1, Fig. 4 indicated by daggers). The 16S rRNA sequences of many types isolated from sugarcane were identical to those isolated from the rhizosphere of the same field. Type 5 represented between 2 and 67% of the sugarcane stem, leaf, and rhizosphere samples across the three fields (Table 1). Types 3 and 27 represented between 4 and 16% of the samples from Field E6. Types 8, 12, and 19 represented between 4 and 33% of the samples from Field A5. Types 3 and 4 represented between 1 and 39% of the samples from Field B7. These results suggested that fenitrothion degraders constituted a part of the plant-associated bacterial community.

**Fig. 7:**
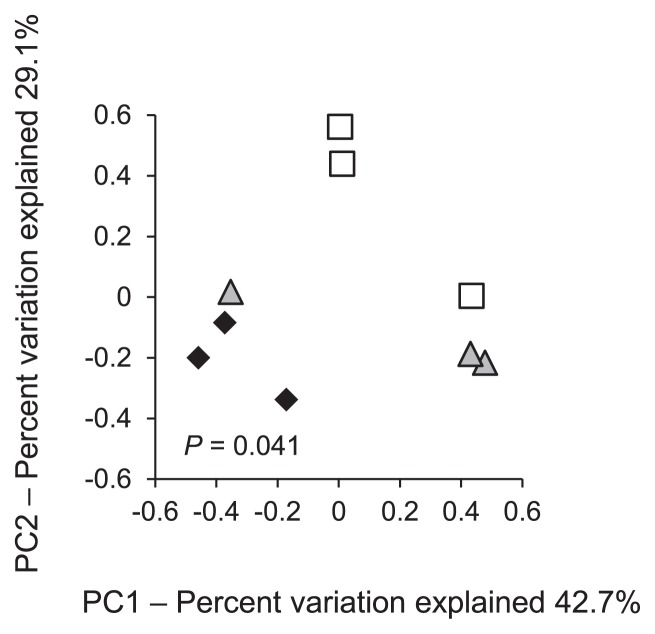
PCoA plots derived from pairwise distances between the communities of degraders in sugarcane stem (open squares), leaf (grey triangles), and rhizosphere (black diamonds) samples.

A PCoA analysis was conducted to determine differences among the degrader communities of the sugarcane stem, leaf, and rhizosphere samples from these fields (Fig. 7). The community compositions from the stem, leaf, and rhizosphere were distinct (AMOVA, *P* = 0.041); however, no significant difference was observed between the stem and rhizosphere (*P* = 0.095), the leaf and rhizosphere (*P* = 0.087), or the stem and leaf (*P* = 0.211). A distinct separation was observed (*P* = 0.041) between the above-ground parts of the plant (stem and leaf) and the rhizosphere. Types 4 and 5 were mainly found in stem and leaf samples while Type 8 was found at a high frequency in the rhizosphere. The degrader communities of the above-ground parts of the plant and the rhizosphere were distinguishable, albeit several identical types of degraders were isolated from the different niches, namely the plant and rhizosphere.

### Identification of fenitrothion degraders associated with *C. saccharivorus*

*C. saccharivorus* were collected from the same sugarcane fields analyzed above. We first attempted to isolate fenitrothion degraders from the dissected symbiotic organ showing the hydrolytic activity of fenitrothion. However, the degraders could not be isolated and cultured from the organ samples using the techniques described in the Materials and Methods. The degraders appeared to become uncultivable in the organ; however, the reason for this was unclear. We previously reported that a single *Burkholderia* species dominated in the midgut tissue of *C. saccharivorus* (20). The lysates of 15 out of 37 samples of symbiotic organ samples showing fenitrothion-degrading activity were succeeded in direct PCR amplification and sequencing of the 16S rRNA gene. All of the obtained 16S rRNA gene sequences were assigned to the SBE group, and 11 sequences were identical to the degraders isolated from the rhizosphere and plant samples in the same field: Types 8 and 32 in Field A5 and Types 8 and 35 in Field B7 (Table 1).

## Discussion

The characterization of symbiotic bacterial distribution patterns at the field scale constitutes an important step in understanding the mechanism of symbiosis in natural environments. Fenitrothion-degrading activity is a preferable trait to monitor the distribution of stinkbug symbionts in the free-living environment. These degraders were not isolated previously from farmers’ (non-experimental) fields. To the best of our knowledge, this is the first study to investigate the distribution of symbiotic *Burkholderia* adapting to fenitrothion in agricultural fields.

The results of this study are consistent with those reported in our microcosm study using various agricultural soils (41); the majority of fenitrothion degraders in sugarcane soils belonged to the genus *Burkholderia*, and degrader density was affected by the extent of exposure to fenitrothion. The SBE group was highly represented among the isolated degraders, constituting 66–100% of the degraders in each soil sample. These results indicated that the SBE group was selected as degraders in response to fenitrothion treatments under field conditions. In other words, sugarcane field soils serve as potential reservoirs for degraders that establish a symbiotic relationship with the stinkbug. An individual host could, via environmental transmission, encounter multiple symbiont genotypes with varying potential host fitness effects (13). The symbiotic *Burkholderia* have been shown to provide *R. pedestris* with benefits, such as increased host fitness and offspring production (22). The survival of stinkbugs confined to the island can be severely threatened by fenitrothion treatments, making fenitrothion resistance a crucial trait. This critical property is provided by various symbiotic *Burkholderia* that have acquired the ability to degrade fenitrothion and that sustain sufficient population sizes for the establishment of symbiosis.

*Burkholderia* species are often found in soil, the rhizosphere, water, as well as various organisms including insects and humans (11). Several studies demonstrated that endophytic *Burkholderia* communities in grapevines and angiosperms originated from the environment (10, 29). Recent studies (23, 29) described overlapping phylogenetic patterns among stinkbug-associated symbionts, leaf endosymbionts (*Candidatus* Burkholderia species), and environmental strains (including fenitrothion degraders). Given that the larval and adult forms of *C. saccharivorus* occupy the basal portion interstices of overlapping leaves at the base of sugarcane shoots (33), we hypothesized that the degraders also inhabited sugarcane and also that transmission to stinkbugs occurred via plants as well as soil. Degraders were successfully isolated from sugarcane and its rhizosphere in fields that had been continuously subjected to fenitrothion treatments. The magnitude of degrader density on leaves was several orders lower than that on the stem or rhizosphere (Fig. 6). This distribution pattern, in which plant sections closer to the ground harbor more bacterial strains, confirmed and extended the findings previously reported for corn (15), pea (14), soybean (28), and sugarcane (31). Over 93% of the degraders isolated from sugarcane clustered within the SBE group. Since the ID_50_ , defined as the amount of symbiont cells required to infect 50% of the tested stinkbugs, was only approximately 80 cells (25), the population size of degraders in the sugarcane stem and rhizosphere appears to be sufficient for symbiotic degraders to encounter their hosts. Seven types of degraders (Types 3, 4, 5, 8, 12, 19, and 27) were prevalent among plants and the rhizosphere. These results strongly suggest that both the plant, especially the lower section of the plant, and rhizosphere serve as environmental reservoirs for symbiotic degraders. Nevertheless, the community composition of degraders in the sugarcane plant was significantly distinct from that in the rhizosphere. This distinction was attributed to the different occupancy patterns of degrader types observed for the plant and rhizosphere. Although our study appears to be unique, the *in situ* niche specificity of rhizobia has already been demonstrated. Sachs *et al.* (36) compared *Bradyrhizobium* species isolated from the root-nodules of wild-grown legumes with those isolated from the root surfaces of the same host individuals. They concluded that the rhizobial genotypes capable of forming root-nodule symbiosis with legume hosts constituted only a small subset of the population present on the root surface. We speculate that a subset of degraders harbors traits that enable them to effectively reach and establish themselves on the plant; alternatively, plants may select these strains if they establish beneficial relationships with sugarcane.

The 16S rRNA sequences of several degraders isolated from the plants and rhizosphere (Types 8, 32, and 35) were identical to the symbionts retrieved from *C. saccharivorus* in the same field (Table 1). This result strongly suggests that the transmission of the soil-born fenitrothion degraders of *Burkholderia* to stinkbugs occurred in natural fields. Garcia *et al.* (17) also compared *Burkholderia* communities in the midgut of stinkbugs (*Alydus* species and *Megalotomus* species) with those in soil and the food crop *Lespedeza cuneata* to identify environmental reservoirs for stinkbug symbionts. A few strains isolated from stinkbugs were closely related, but not identical, to those found in soil and on plants. Our results strongly indicate that the degraders of the SBE group may have the ability to occupy three different environments: the rhizosphere, plant, and stinkbug. On the other hand, *C. saccharivorus* larvae were detected on the lower stem section, several centimeters above the cut end (Fig. S1), when sampling sugarcane, indicating that larvae travel around the plant and soil. In contrast, soil particles were often observed on the interstices of overlapping leaves on the lower portions of stem samples. Therefore, *C. saccharivorus* may also have many opportunities to encounter symbiotic degraders derived from the soil particles on sugarcane. More information regarding the ecology of *C. saccharivorus* in its local environment is required to better understand the process involved in the transmission of the symbiotic fenitrothion degraders of *Burkholderia* species.

## Supplementary Information



## Figures and Tables

**Table 1:** Number of degraders isolated from sugarcane and rhizosphere samples in Fields A5, B7, and E6

Type	Field No.											

	A5				B7				E6			
		
	Rhizosphere	Shoot	Leaf	Stinkbug*	Rhizosphere	Shoot	Leaf	Stinkbug*	Rhizosphere	Shoot	Leaf	Stinkbug*
The SBE group												
3	3	2			1	2	9		1	3	1	
4		98	7		3	10	1		4	4		
5	2	3	2		2	2	5		1	7	8	
8	5	6	14	3	46	3		3	10			
11		3	2				2		2	1		
12	1	9	8		1		1		1	1		
19	1	14	11									
22		2	1				2			1		
24		1										
25		10										
27	2				1				4	2	1	
28							2					
32		1	1	2								
34		1			2						1	
35				1			1	2				
36					1							
38									1			

Others												
6									1			
20	1				5	5						
26						11						
29					2							
30					1						1	
31					2							
33					1					1		
37		1										

* PCR products from the lysates of symbiotic organ samples showing fenitrothion-degrading activity.
