# Supplementary material for: Insecticide-Degrading Burkholderia Symbionts of the Stinkbug Naturally Occupy Various Environments of Sugarcane Fields in a Southeast Island of Japan
Source: Microbes Environ. 2014 Dec 27;30(1):29–36. doi: 10.1264/jsme2.ME14124 (PMC4356461; doi:10.1264/jsme2.ME14124)
Supplement: Supplementary file 1 [file 30_29_s1.pdf]

Table S1 Insecticide treatment history and soil chemical characteristics of sugarcane fields

Field No.	Insecticide treatment			pH		C/N		NH_4^+-N (μg/g dry soil)		NO_2^--N (μg/g dry soil)		NO_3^--N (μg/g dry soil)		P (μg/g dry soil)	
	2010	2011	Category**	surface	subsurface	surface	subsurface	surface	subsurface	surface	subsurface	surface	subsurface	surface	subsurface
A1	o*	o, i*	O	5.68	4.79	6.5	7.4	230.4	127.1	0.07	0.08	80.6	86.8	97.8	99.6
A2	u*	o	O	6.32	5.40	7.8	7.0	96.1	29.8	0.11	0.13	54.1	81.4	58.4	91.4
A3	o	n*	O	5.40	4.85	7.8	7.0	11.8	3.4	0.09	0.08	9.9	27.1	66.6	69.8
A4	o, i	n	O	4.54	4.30	9.1	7.7	31.5	4.8	0.14	0.20	12.8	38.6	64.5	68.8
A5	u	f*	F	4.64	4.47	8.6	7.9	14.4	18.9	0.13	0.18	6.1	4.5	56.3	65.4
B1	i	i	I	6.21	6.54	7.8	8.6	40.5	6.0	0.08	0.09	180.7	52.3	77.3	80.5
B2	n	i	I	4.32	4.33	7.2	6.7	237.2	296.8	0.09	0.09	60.7	35.4	82.3	89.8
B3	f, o, i	o, i	F	5.84	6.14	8.8	8.00	9.0	6.7	0.09	0.11	2.3	18.5	57.6	71.7
B4	f	f, i	FF	5.51	4.87	8.8	9.4	16.9	1.7	0.10	0.11	8.1	20.3	69.2	78.6
B5	f	f	FF	4.23	3.60	7.3	7.4	229.6	5.1	0.11	0.19	117.0	185.9	120.7	112.8
B6	f	f	FF	4.17	3.71	9.3	7.6	188.5	3.4	0.04	0.24	58.8	34.0	96.1	125.6
B7	f	f, i	FF	5.10	4.19	6.5	7.8	303.8	9.8	0.15	0.14	11.5	266.2	69.5	81.1
C1	n	n	I	6.36	6.58	8.9	7.9	7.3	5.0	0.08	0.09	0.2	0.2	58.9	63.3
C2	i	i	I	5.31	4.97	8.65	9.0	83.1	4.1	0.10	0.11	81.2	53.4	77.8	84.5
C3	o	o, i	O	6.70	6.75	13.9	14.3	60.2	5.7	0.13	0.10	109.2	64.8	59.7	58.9
C4	f	f	FF	5.78	5.23	9.0	7.6	24.2	15.8	0.12	0.16	6.1	2.1	48.8	60.6
C5	n	n	I	6.31	5.87	6.0	6.1	399.2	229.6	0.14	0.16	212.4	267.9	172.5	126.2
D1	f	f, o	FF	4.77	4.09	6.9	7.3	119.8	13.2	0.11	0.07	36.4	280.2	60.3	88.4
D2	u	f, o	F	5.89	5.44	6.6	7.9	163.2	28.6	0.1	0.09	102.3	84.4	100.4	83.7
D3	o	o	O	4.79	4.56	9.3	9.4	32.7	6.4	0.08	0.16	50.3	43.7	79.5	79.8
E1	f	f	FF	4.88	4.58	8.1	8.0	18.6	13.8	0.07	0.06	17.9	19.2	62.6	110.6
E2	f	o	F	5.20	4.58	5.9	6.7	352.4	43.4	0.10	0.13	68.5	55.8	118.6	112.6
E3	o, i	i	O	4.64	3.63	6.4	7.6	440.4	3.3	0.07	0.14	38.1	277.7	138.7	120.8
E4	f, i	f, i	FF	5.32	4.77	8.7	6.7	169.0	149.9	0.12	0.08	198.2	113.4	102.1	91.1
E5	n	f, i	F	7.62	7.62	11.8	10.4	78.0	170.2	0.17	0.12	30.8	171.0	45.3	52.0
E6	f	f, i	FF	6.08	6.32	7.9	8.5	39.4	32.9	0.12	0.12	9.7	6.4	61.7	63.0
E7	o	n	O	7.89	7.79	18.3	16.5	20.4	3.3	0.12	0.18	21.4	41.5	63.6	107.8

* f, fenitrothion; o, other organophosphorus insecticides; i, non-organophosphorus insecticides; u, unknown; n, none.
**For statistical analysis, the sugarcane fields were categorized into four based on insecticide treatment history. FF, 2–year continuous treatment of fenitrothion in 2010 and 2011; F, 1–year treatment of fenitrothion in 2010 or 2011; o, 1– or 2–year treatment of other organophosphorus insecticides; I, both 1– or 2–year treatment of non-organophosphorus insecticides and no treatment of insecticides.

Table S2 Insecticide treatment history in 2012 and 2013

Field No.	2012	2013
A5	f* (2)**	n
B6	f (1), i (1)	n
B7	f (4)	f (3)
C4	f (1)	n
D3	f (1), o (1)	f (1)
E5	o (1)	n
E6	f (>2)	f (2)

* f, fenitrothion; o, other organophosphorus insecticides; i, non-organophosphorus insecticides; n, none.
** Times of insecticide treatment were represented in parentheses.

Table S3 Number of isolates of Types 8, 12, and 19 in the island

Sample	Number of isolates		
	Type 8	Type 12	Type 19
A5	3		4
A5L*	4		1
B2			
B2L	1		1
B3			
B3L	2		
B4	23	4	9
B4L			
B6	1	2	10
B6L			1
B7	26	10	
B7L	13		2
C2	12		
C2L	8	2	
C4	1	1	
C4L			
D3	1		
D3L			
E1	5		6
E1L			
E2	2		
E2L			
E4	4		
E4L			
E6			
E6L	1		

*If the sample was collected from subsurface soil, the subscript "L" is placed after the field number.

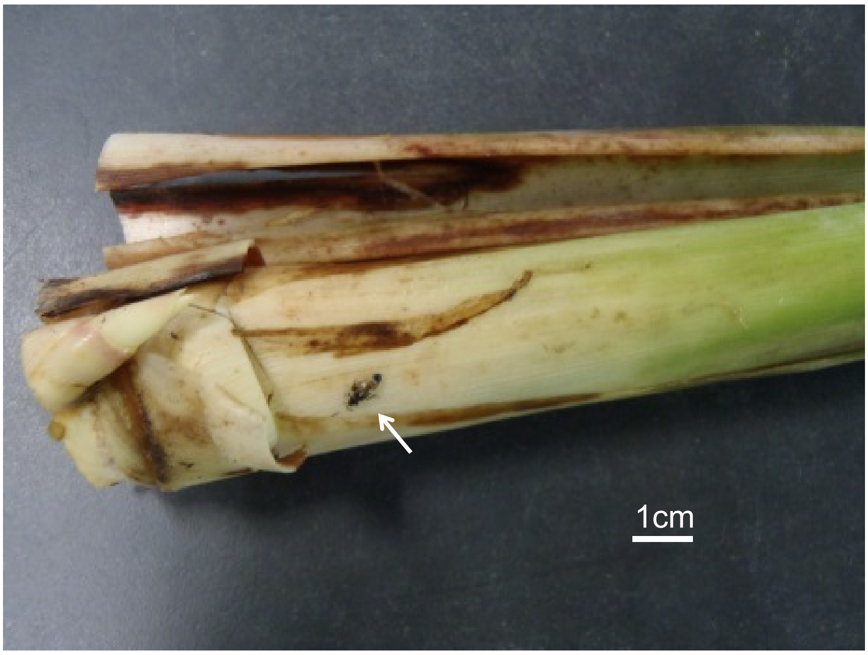

Fig. S1 Photograph of a larva of *C. saccharivorus* on stem sample (represent by an arrow).
